# Determination of Correlations between Melt Quality and the Screw Performance Index in the Extrusion Process

**DOI:** 10.3390/polym15163427

**Published:** 2023-08-16

**Authors:** Dorte Trienens, Volker Schöppner, Robin Bunse

**Affiliations:** Kunststofftechnik Paderborn, Paderborn University, 33098 Paderborn, Germany; volker.schoeppner@ktp.upb.de (V.S.);

**Keywords:** extrusion, screw performance index, melt quality, simulation

## Abstract

For polymer-processing extruders, designing screws via analytical computational models is helpful for reducing experimental costs. However, the current simulation programs cannot predict the melt quality at the screw tip with sufficient accuracy. There are a number of definitions of melt quality in the literature. This paper will review some of these definitions and present how melt quality can be assessed in subsequent work. In this paper, both the thermal and material homogeneity of the melt quality are examined for correlations with the screw performance index. If correlations exist with the screw performance index determined from direct experimental measurement data, these can be used as target values for developing a melt quality prediction model.

## 1. Introduction

In the field of polymer processing, polymers are plasticized so that they can be further processed into end products or semi-finished products. Polymers are often processed on screw machines, some examples of which are extruders and injection-molding machines. Each plastic is processed at least once on a single-screw extruder on its path from polymerization to becoming an end product [1]. The cost efficiency of the processing operation is usually an important factor due to a desire to maximize profits [2]. This depends not only on the processing parameters of the converting process, such as the screw speed and barrel temperatures, but also on achieving the maximum possible plasticizing and homogenizing performance of the extruder while still maintaining sufficient product quality [3]. The process parameters are often manually specified by the machine operator and can possibly be optimized so that an increase in throughput can be achieved while maintaining identical product quality. However, the maximum possible plasticizing and homogenizing performance is already limited by a non-optimal extruder design [4]. This results in a process with limited throughput. In such a process, the possible output rate of the extruder is reduced compared to an optimized process, resulting in economic losses [5].

For this reason, the design of the extruder’s hardware, especially the extruder screw, plays a particularly important role in the subsequent cost efficiency. Since the process of screw design should not involve the use of prototypes for reasons of cost and time, the screw designer is left only with empirical values and simulations as reference points [6]. At present, simulations cannot be used to make reliable statements about the expected quality of the product [5]. Product and melt quality can only be determined through experimental studies and as functions of various process characteristics such as wall thickness, pressure variations and the degree of melting [7]. Current simulation programs output temperature and pressure curves along the length of the screw, but these are not interpreted [8]. These dependencies, which need to be investigated, can be summarized in a mathematical model. This model can then be applied to simulations in order to draw conclusions about the expected product quality on the basis of these simulations.

**Figure 1:**
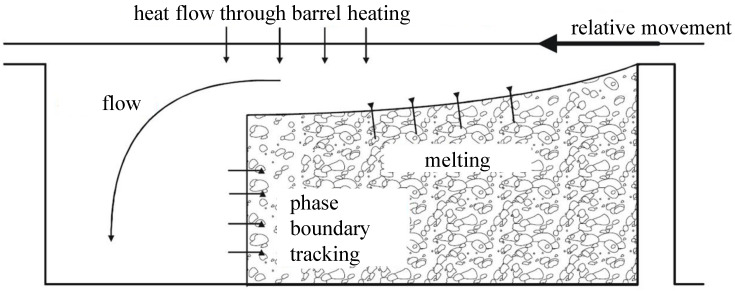
Tadmor model [9].

In order to simulate the extrusion process, it is necessary to theoretically describe the melting process in the extruder. Tadmor was the first to develop a mathematical model of the melt zone. Figure 1 shows the Tadmor melting model. It consists of a solid bed, a melt pool and a melt film between the barrel and the solid bed [9]. The granule begin melting when the high temperature on the cylinder wall creates an initial melt film. When this “melting” has progressed far enough, the melt accumulates on the active flank. As the melt continues to flow through the screw, the melt pool expands until the end of the screw is completely molten [10]. In the Tadmor model, the melt pool increases in size because the material melting at the interface is transported into the melt pool by the crossflow. As the screw length increases, the melt pool widens and the proportion of melted material increases. Toward the end of the melt, the remnants of the solid bed break up and float like islands in the surrounding melt [10].

The aim of this research is to establish correlations between process parameters, melt quality and product quality. The correlations to be used must first be investigated and evaluated to ensure their suitability for making predictions in a simulation model. Once suitable correlations have been determined, they are to be used to predict melt quality by means of the process parameters.

This quality criterion should already be calculable and usable for the screw feeder. In this way, a targeted screw design can lead to improved process behavior and thus to higher throughput with consistent melt quality.

### 1.1. The Most Common Screw Concepts

In the following section, the three screw concepts used in this work are presented. Shear and mixing elements in combination with a three-section screw, barrier screws and three-section screws are investigated.

#### 1.1.1. The Three-Section Screw and Barrier Screw

**Figure 2:**
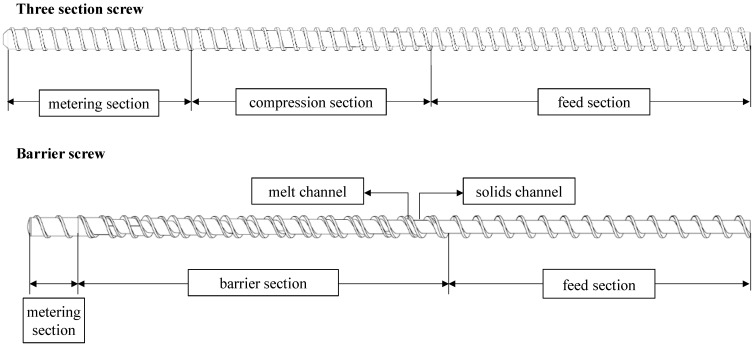
Schematic of a barrier screw.

Figure 2 shows the structures of a three-section screw and a barrier screw. A common screw design is the three-section screw, which can process a wide range of material types and is therefore universally applicable [11]. As the name suggests, this type of screw consists of three sections, each of which performs a different task. These sections are called the feed section, the compression section and the metering section according to their function [12]. Within the first section, the feed section, the solid polymer is taken up as granules by the hopper. At a constant passage depth, the material is conveyed, compacted and preheated for the next zone [13]. The driving force in the direction of the screw tip arises from the friction between the stationary barrel and the polymer granules resulting from the rotary motion. Friction between the polymer and the screw, on the other hand, would inhibit conveyance, which is why the screw surface is designed to be frictionless [14]. The compression section transfers the polymer to the molten state. A large amount of heat is added to the melt via viscous dissipation. This occurs due to the shearing of the material by the screw’s rotation [15]. The passage depth within this section decreases constantly, which compresses the plasticized molding compound. This results in an increase in pressure and causes the material’s contact with the heated cylinder wall to increase, which also contributes to the melting process [4]. The length of this zone is influenced by the material properties, screw geometry and processing conditions [16]. Within the metering section, the melt is homogenized, mainly thermally but also partly in terms of material, and brought to the desired processing temperature at a constant transition depth [14]. This is where the necessary pressure is built up in conventional extruders to overcome the resistance for the die [17,18,19].

Barrier screws are used to increase throughput. The increase in throughput is made possible by the increased energy input [2]. A barrier screw has two flights: a main flight and a barrier screw flight. The purpose of the screw is to separate the solid material from the already molten material. The barrier screw effectively separates the solid bed from the melt. To prevent the solid from mixing with the melt, the distance between the barrier bar and the barrel is very narrow so that only the already-melted polymer can pass through the gap and granules cannot [3,15]. Along the length of the screw, the volume of the solid decreases, while the volume of the melt increases. The channel depths and flight widths of both channels do not have to be identical, which is why an adjustment of the volume flow or the channel width is possible [20]. In contrast to the previously described three-section screw, larger channel depths and thus higher specific throughputs are possible due to the high energy input [20].

#### 1.1.2. Shearing and Mixing Section

**Figure 3:**
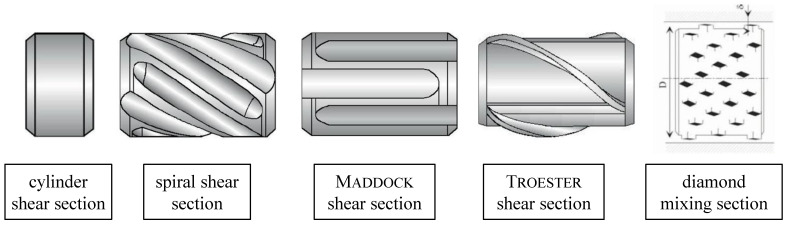
Schematics of shear and mixing sections [10].

For good product quality, the melt must be in a materially and thermally homogeneous state while it is being processed in the die. The temperature should be constant over the entire cross-section, and there should be no differences in concentration between additives and fillers. To ensure this, shear and mixing sections are used at the screw tip. Usually, the shear section is mounted on the screw before the mixing section. [7]. The function of the shear section is to guide the melt over a long, narrow flight close to the inner wall of the cylinder. In this way, particles that have not yet melted are retained or broken up and melted by the high shear forces that occur. In addition, the melt is thermally homogenized by its close contact with the cylinder wall. The simplest design is the cylinder shear section, as shown in Figure 3. More complex designs, however, are the spiral shear section, the Maddock shear section and the Troester shear section [7]. Mixing elements, on the other hand, split the melt flow several times and recombine it. Flow-dividing elements are placed in the path of the flow so that the flow is mixed and homogenized. The more strain components a flow has, the more effective a mixing section is [7]. The shapes of the mixing section are usually not optimal for melt conveying, resulting in a loss of pressure. To improve homogeneity, a reduction in throughput and an increase in melt temperature are accepted. However, if the mixing sections are designed well, the pressure loss and temperature increase are limited despite good mixing efficiency. In recent years, the diamond mixing section has gained acceptance [20]. 

### 1.2. Quality Criteria of Polymer Melts

In order to ensure a suitable polymer melt flow for processing, the melt can be assessed in terms of its quality on the basis of various criteria. Good melt quality is indicated by a uniform melt temperature distribution across the cross-section (thermal homogeneity), as well as the uniform mixing of the melt (material homogeneity) at a constant temperature and back pressure over time [21]. Accordingly, care must be taken to ensure low-pulsation conveying. In addition, other factors, such as the degree of melting, the pressure curve, material damage and energy efficiency, have an influence on the quality of the melt. Processing must continue to be carried out below the thermal, chemical and mechanical damage limits [7].

#### 1.2.1. Degree of Melting and Melt Temperature

Melting is an essential process in extrusion in which the polymer is completely converted into a molten state. If the energy input is too low and the melting process is therefore incomplete, solid particles will form within the melt and pass through the extrusion die. These solid particles, in turn, have a negative effect on the quality of the end product which is clearly visible, for example, when producing a film. As a rule, more solid particles occur when the screw speed and the associated throughput are increased [6]. Accordingly, the optimum melt temperature is reached when the material has melted completely. If the polymer melt is heated further, an unnecessarily large amount of energy is consumed, both during the heating and cooling of the product. In addition, the main valence bonds can be separated, which leads to the destruction of material when the decomposition temperature is reached [18].

#### 1.2.2. Material and Thermal Homogeneity

It is necessary to ensure the melt is sufficiently homogenous, both materially and thermally, in the extrusion process. Thermal homogeneity is important for ensuring consistent material performance over the entire cross-section of the die, especially with respect to flow behavior. If this is not the case, different exit speeds can occur at the die exit, which can lead to insufficient dimensional accuracy [21]. It is also necessary to ensure sufficient material homogeneity, for example, to distribute fillers or colorants evenly in the extrudate, in order to ensure that the mechanical and decorative properties are uniform throughout the extrudate at each location. Insufficient homogeneity can therefore lead to product defects. It has been shown that homogeneity generally decreases at higher throughputs due to short residence times and subsequent complete melting. Therefore, the aim should be to achieve high levels of both material and thermal homogeneity [22].

#### 1.2.3. Fluctuations in Pressure and Temperature

It is important that the melt at the end of the screw has as uniform a temperature as possible across the entire cross-section to ensure thermal homogeneity. One means of confirming this is to measure the temperature at different points in the cross-section and then calculate the standard deviation. A higher standard deviation indicates greater differences between the temperatures across the cross-section and signifies worse thermal homogeneity. A common method of determining the temperature across the cross-section is to use a measuring blade or measuring bar [23]. The device is mounted between the screw tip and the die to measure the radial temperature profile of the melt. The measuring blade is used to determine a characteristic value based on temperature sensors that are immersed in the melt at different depths. Subsequently, a weighted average temperature of the melt can be calculated using the pure pressure flow in the flange of the measuring blade. Here, it is necessary to define a weighting factor for each temperature measuring point, which is also dependent on the material used [21].

#### 1.2.4. Pressure Curve or Maximum Pressure

Statements about the quality of the melt can also be made on the basis of fluctuations in the stationary pressure and the stationary temperature over time. A fluctuation in the pressure signal can indicate a variation in the throughput over time. This can lead to variations in the product’s geometry, for example. Typical causes of fluctuations in the back pressure are deep-cut metering zones, fluctuating throughputs during infeed or screw plugging. In addition to these effects, the pressure fluctuation also serves as a measure of the homogeneity of the melt [15]. As a result, unmelted particles, a fluctuating distribution of fillers or even temperature fluctuations can become visible in the pressure curve. The reasons for the occurrence of pressure fluctuations also lead to temperature fluctuations. In principle, pressure and temperature fluctuations have opposite effects. For a high-quality melt, therefore, temperature fluctuations should also be reduced to a minimum [24].

In addition to the back pressure fluctuation, the pressure gradient across the screw is relevant to achieving good extrudate quality. The screw should build up pressure continuously and have as few areas with negative pressure gradients as possible. This means that the air in the granulate is degassed in the direction of the hopper and that no air bubbles occur within the melt. In addition, the pressure flow, which counteracts the drag flow, improves the mixing effect and consequently the homogeneity of the melt [24].

#### 1.2.5. Material Damage and Degradation

A typical challenge in extrusion is material damage due to molecular weight degradation [21]. During extrusion, decomposition usually occurs through a combination of thermal, mechanical and chemical stresses [5]. Thermal material degradation involves the splitting of the carbon–carbon bonds of the polymer molecules under high temperatures, for which an activation energy is required. As the temperature increases, the rate constant of this degradation reaction increases exponentially, resulting in an enhanced degradation reaction that can be detected. Temperature is not the actual cause of degradation but merely a factor that enhances it. Cleavage reduces the average molar mass of the polymer molecules, leading to changes in their properties. Cleavage occurs along the polymer chain in a statistically distributed manner, resulting in a lower overall decrease in the average molecular mass [5,21,25]. 

Reductions in molecular weight are usually due to changes in the flow’s behavior and therefore viscosity. Therefore, it is a good idea to determine the melt flow rate (MFR) in the melt index analyzer [26]. This single numerical value indicates the volume or mass of a plastic sample under a predefined temperature and pressure flowing out of a standardized nozzle in ten minutes [27]. The MFR value is given for each string in g/10 min. Subsequently, the average value is formed from this. By subsequently weighing the extruded strand, the MFR value can be calculated.

#### 1.2.6. Energy Efficiency

The requirements placed on an extrusion line have changed significantly in recent years. In the past, the output was almost exclusively the decisive criterion, whereas today, the efficiency and flexibility of the systems play increasingly important roles [10]. A high throughput with a constant machine size results in reductions in both acquisition and operating costs [23]. Screws with lower pressure peaks have longer service lives due to less mechanical wear. In addition to purchase and installation costs, an extrusion line also incurs ongoing energy costs. It is therefore desirable for an extrusion line to operate as efficiently as possible and for the majority of the energy invested to be incorporated into the polymer. Possible energy losses arise, for example, from electrical losses, the use of gears with low efficiency or the cooling of the barrel or screw [21].

### 1.3. Screw Performance Index

According to Dörner [21], the criteria described above can be summarized in the screw performance index (SPI), introduced by him for the screw configurations under consideration. According to Equation (1), this results in a single numerical value between 0 and 1 which can be assigned to the respective screw configuration. The larger the SPI, the better the performance of the screw. 

Each criterion is divided into five classes from 0 to 4. Class 0 is the best class. In terms of the degree of melting, class 0 represents a completely melted melt. As the class increases, the number of solid particles at the die exit increases. The degree of melting is also a necessary condition for calculating the SPI. Beginning already from class 2, defects accumulate so regularly that the melt is inadmissible for further processing and the SPI drops directly to a value of 0. It should be noted that this is a subjective observation since the evaluation of the degree of melting cannot be measured directly but is based on empirical values. Analogous to the degree of melting, the maximum and minimum values of each criterion are analyzed for each combination of material, screw and process point. Between these two values, the five equal classes are spanned, and the screws and process points are then assigned to these classes. Each class is given a character, c, and its respective characteristic value in the index. In the next step, an average value is calculated for all classes. With the exception of the difference between the max. pressure and back pressure *Ck_Druck_*, all criteria are weighted with the factor 1. Only the difference between max. pressure and the back pressure *Ck_Druck_* is weighted with a factor of 0.5. In addition to the degree of fusion, the averaged melt temperature *T_GM_* is also a necessary condition. This must lie between specified material-dependent limits. If both conditions are fulfilled, the SPI can be determined according to Equation (1) [21].

(1) $$\begin{array}{c} SPI=\left(-\frac{C_{k_{melt}}+C_{k_{\Delta p_{stern}}}+0.5*C_{k_{Druck}}+C_{T_{GM}}+C_{k_{T_{GM}}}+C_{k_{tH}}+C_{k_{Eff}}+C_{k_{MFR}}}{7.5*4}+1\right),\\ C_{k_{melt}}>2\to SPI=0\\ T_{GM}<T_{Grenz_{min}}\cup \;T_{GM}>T_{Grenz_{max}}\to SPI=0 \end{array}$$

*SPI*: Screw performance index;
$T_{Grenz}$: Minimum and maximum processing temperatures of the polymer;
$Ck_{melt}$: Degree of melting;
$Ck_{\Delta p_{stern}}$: Back pressure fluctuation;
$Ck_{Druck}$: Difference between the max. pressure and the back pressure;
$CT_{GM}$: Melt temperature;
$Ck_{T_{GM}}$: Temperature fluctuation;
$Ck_{tH}$: Thermal homogeneity;
$Ck_{Eff}$: Degree of efficiency;
$Ck_{MFR}$: Change in the MFR value.

## 2. Materials and Methods

The following chapter presents the materials and experimental equipment used in this work. In addition, the evaluation methods used to determine melt quality are presented.

### 2.1. Machine Configuration

**Figure 4:**
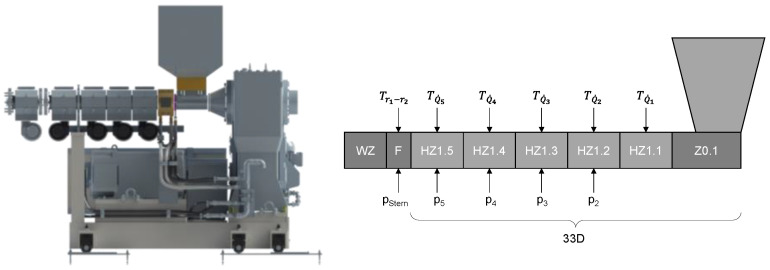
Schematic extruder setup with measuring technology, consisting of a cooled feed section (Z0.1), barrel-heating sections (HZ1.1–HZ1.5), a flange (F), and a die (WZ).

The investigations were carried out on a single-screw extruder from Reifenhäuser Extrusion Systems GmbH (Troisdorf, Germany), model RH034-45-28D/HS, in the technical center of Kunststofftechnik Paderborn (Figure 4). The extruder’s inner barrel diameter is 45 mm, with a process length of 33D. The extruder design consists of a cooled feed section (Z0.1) and five barrel-heating sections (HZ1.1–HZ1.5). A flange (F) is located between the barrel and the die (WZ). The barrel is equipped with four pressure sensors (p_2_–p_5_, Gefran, Seligenstadt Germany, IMPACT IEC 1000 bar) and five temperature sensors (T_Q1_–T_Q5_, Gneuss, Bad Oeynhausen, Germany, type TF-MX) along its entire length. After the last heating zone of the extruder, a temperature-controlled flange is mounted in which the pressure, pstar, is measured and the radial temperature profile at the screw tip is recorded. For this purpose, as described by Chung [15], five temperature sensors are provided in the flange, which protrude into the screw channel in a star shape at different depths (5, 10, 15, 20 and 22.5 mm, Gneuss type, Bad Oeynhausen, Germany, TF-CX). The die is a wide slot die from COLLIN Lab & Pilot Solutions GmbH, Maitenbeth, Germany. The melt is distributed in a rectangular cross-section over a width of 300 mm with the aid of a coat-hanger distributor. The measurement data were recorded using a Q. Gate measurement system from Gantner Instruments Tests & Measurement GmbH, Lauf an der Pegnitz, Germany. The signal was recorded at a sampling frequency of 10 Hz as this allows the temperature and pressure fluctuations to be mapped with sufficient accuracy.

### 2.2. Investigated Materials

Two different polymers from the company LyondellBasell were used for the experimental investigations. The low-density polyethylene (LDPE) is called Lupolen 2420D and the name of the polypropylene is HP420M. The Carreau parameters at 230 °C for both materials are listed in Table 1. The calculation of viscosity using the Carreau approach is shown in Equation (2). For the investigations of material homogeneity, color master batches were added to the base material. The Black 335 PP pigment was compounded into Moplen 420M from Argus Additive Plastics GmbH, and the Hostalen CRP 100 Black was compounded into the Lupolen 2420D. In order to compare the results of the grey value analysis, 0.4% of the pigment by mass was added to the PP and 7.5% by mass was added to the PE.

(2) $$\eta =\frac{A}{{\left(1+B\dot{\gamma }\right)}^{C}}$$

η: Viscosity;
$\dot{\gamma }:$ Shear rate;
A: Zero viscosity;
B: Reciprocal transition shear rate;
*C*: Slope of the shear thinning viscosity (1 − *n*);
*n*: Flow exponent.

**Table 1:** The melting point, zero viscosity, reciprocal transition shear rate, the slope of the shear thinning viscosity and hte solid density of Moplen 420M and Lupolen 2420D at 230 °C.

Material	Melting Point [°C]	Zero Viscosity *A* [Pa∙s]	Reciprocal Transition Shear Rate *B* [s]	Slope of the Shear Thinning Viscosity C [-]	Solid Density [kg/m³]
Moplen 420M (PP)	167	1390	0.0633	0.678	0.9
Lupolen 2420D (PE)	114	19,335	1.57699	0.633	0.918

### 2.3. Screw Concepts Investigated

**Figure 5:**
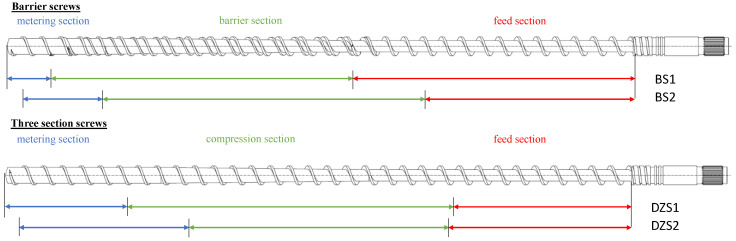
Schematic screw configurations of the two barrier screws (BS1 and BS2) and three-section screws (DZS1 and DZS2).

In the investigations, three different screw concepts were examined, each with two different configurations that differ slightly in their geometry. The aim was to determine the influence of changes in screw geometry within a screw design on both the thermal and material homogeneity. The concepts investigated were the barrier and three-section screws without shear and mixing parts. The schematic screw configurations of the two barrier screws and three-section screws are shown in Figure 5. In addition, a standard three-section screw was combined with two different shear parts and a mixing part. The two barrier screws investigated, “BS1” (30.89 L/D) and “BS2” (30 L/D), have almost the same length. The feed section of BS1 at 13.5 L/D is significantly longer than that of BS2, with 10 L/D. The typical barrier zones with melt and solid channels differ by only 0.4 L/D. At 2.1 L/D, the metering zone of BS1 is significantly shorter than that of BS2 (3.8 L/D). The two three-section screws (DZS1 and DZS2) are also comparable in length (32.89 L/D and 31.22 L/D). The differences between the two screw geometries lie in the lengths of the melting zone and the metering zone. The melting zone of screw DZS1 is 2.2 L/D longer than that of DZS2. The metering zone of DZS2 is correspondingly longer. In addition, the flight depth of the DZS2 screw is cut significantly deeper than that of the DZS1 screw. The standard three-section screw has a 7.22 L/D long feed section, a 10 L/D long compression section and a 6 L/D long metering section. Two different shear parts are then mounted. First, the influence of a Maddock shear section (SM1), and second, a spiral shear section (SM2) in combination with the same diamond mixing section are investigated. This results in a total length of 28.44 L/D. The temperature profiles for the two materials are shown in Table 2. The temperature profile for the Moplen 420M on the barrier screws was selected according to Dörner [21]. Barrier screws often tend to clog due to the closed inlet if the plastic is not sufficiently melted up to the barrier zone. For the other temperature profiles, the manufacturer’s specifications were followed. 

### 2.4. Investigation Plan

Table 3 shows the investigation plan. The material, the screw concept and two screw geometries as well as the specified throughput were varied. In the investigations, the thermal homogeneity was determined on the basis of the standard deviation of the weighted average melt temperature, and the material homogeneity was determined on the basis of the standard deviation of the grey value. The weighted average melt temperature was determined in the temperature measuring star. Due to the different flow profiles in the channel cross-section, the temperatures of the temperature sensors projecting into the channel at different depths were multiplied by weighting factors. This resulted in the weighted average melt temperature. The standard deviation was determined in order to assess the thermal homogeneity. The material homogeneity was evaluated by means of the dead-stop test and the subsequent grey value analysis.

### 2.5. Evaluation Methods

As part of the investigations, the temperature and pressure curves recorded by the measuring computer were evaluated over the entire screw length and over time in the measuring flange. In addition, a dead-stop test was carried out in each case, and the thin section sample was subjected to a gray value analysis. The absolute values of the temperatures and gray values were not suitable due to the different temperature profiles and the different gray tones of the different materials. To characterize the material homogeneity, the averaged frequency distribution of the gray values is determined. Figure 5 shows two samples for the gray value analysis as examples. Deep white and black areas in the thin slice indicate that the base material has not mixed with the master batch, suggesting poor melt homogeneity in the material. In the gray-level analysis, the gray level of each pixel was evaluated on a scale from 0 (black) to 255 (white). The arithmetic mean for each gray value was calculated from the five thin slices made per operating point. Subsequently, the mean gray value $\bar{G}$ of the averaged frequency distribution was calculated as shown in Equation (3).

(3) $$\bar{G}=\frac{1}{P}\;\overset{255}{\underset{i=0}{\sum }}g_{i}p_{i}$$

$\bar{G}$: Average gray value;
P: Total pixels;
$g_{i}:$ Gray value;
$p_{i}:$ Number of pixels with this gray value.

**Figure 6:**
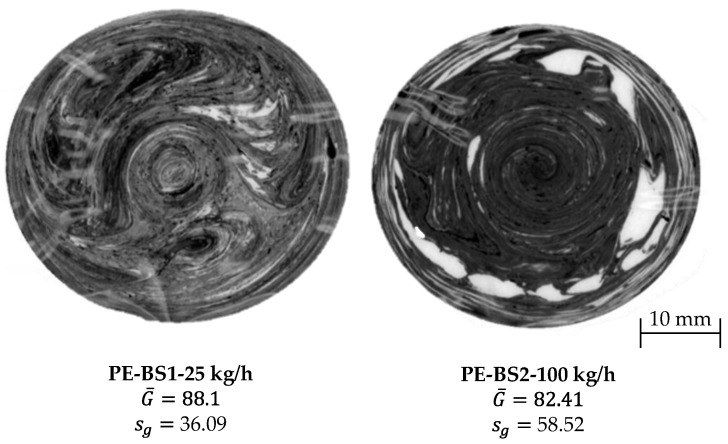
Schematic extruder setup with measuring technology.

Using this mean, the variance *s*^2^ and the standard deviation *s_g_* were calculated. Both values represent the deviation of the individual gray values from the previously calculated mean. The goal of good blending is to achieve the lowest possible standard deviation. In Figure 6, there are significantly fewer white and black areas in the left thin slice compared to the right. This observation is also reflected in the standard deviation of the gray value.

From the equation for the flow velocity of a power-law fluid in the axial direction of a pipe cross-section (compare Equation (4), [28]), the mean flow velocity in a circular ring can be determined under the assumption of an incompressible fluid (compare Equation (5)).

(4) $$v_{z}\left(r\right)=\frac{n*R}{1+n}\;\;{\left(\frac{R*\;\Delta p}{2*K*L}\right)}^{\frac{1}{n}}*\;\left[1-{\left(\frac{r}{R}\right)}^{1+\frac{1}{n}}\right]$$

$v_{z}\left(r\right):$ Flow velocity in the axial direction;
r: Considered radius;
n: Flow index;
K: Consistency factor;
R: Inner radius of the pipe cross section;
L: Length of the pipe cross-section;
Δp: Pressure difference.

(5) $$\bar{v_{z_{i}}}=\frac{n*R}{1+n}\;\;{\left(\frac{R*\;\Delta p}{2*K*L}\right)}^{\frac{1}{n}}*\left[1-\frac{2*\;R^{2}}{\left(r_{i-1}{}^{2}-r_{i}{}^{2}\right)}*\;\frac{1}{\left(3+\frac{1}{n}\right)}*\;\left({\left(\frac{r_{i-1}}{R}\right)}^{3+\frac{1}{n}}-{\left(\frac{r_{i}}{R}\right)}^{3+\frac{1}{n}}\right)\right]$$

$\bar{v_{z_{i}}}:$ Mean flow velocity in the circular ring;
$r_{i-1}$: Outer diameter of the circular ring;
$r_{i}:$ Inner diameter of the circular ring.

Using the area of the circular ring (Equation (6)) and the total volumetric flow rate of a power law fluid in a circular cross-section (Equation (7), [28]) results in the calculation of the weighting factor *X_i_* (Equation (8)).

(6) $$A_{i}=\pi \;*\;\left(r_{i-1}{}^{2}-r_{i}{}^{2}\right)$$

$A_{i}:$ Area of the circular ring.

(7) $$\dot{V_{ges}}=\frac{\pi *\;n*\;R^{3}}{1+3*n}*\;{\left(\frac{R*\;\Delta p}{2*K*L}\right)}^{\frac{1}{n}}$$

$V_{ges}:$ Total volume flow.

(8) $$X_{i}=\frac{A_{i}\;\bar{v_{zi}}}{\dot{V_{ges}}}=\frac{\left(1+3n\right)}{\left(1+n\right)}\;\left[{\left(\frac{r_{i-1}}{R}\right)}^{2}-{\left(\frac{r_{i}}{R}\right)}^{2}\right]-\frac{2n}{1+n}\;\left[{\left(\frac{r_{i-1}}{R}\right)}^{3+\frac{1}{n}}-{\left(\frac{r_{i}}{R}\right)}^{3+\frac{1}{n}}\right]$$

$X_{i}$: Variance;
$A_{i}:$ Area of the circular ring;
$\bar{v_{zi}}$: Mean flow velocity in the circular ring;
$V_{ges}:$ Total volume flow;
n: Flow index of the power law;
$r_{i}:$ Inner diameter of the circular ring;
$r_{i-1}:$ Outer diameter of the circular ring;
R: Inner radius of the pipe cross-section.

The weighting factor depends on both the material (flow index *n*) and the geometric design of the measuring flange (radii *r_i_*, *R*). The average melt temperature *T_GM_* is calculated using Equation (9).

(9) $$T_{GM}=\overset{N}{\underset{i=1}{\sum }}X_{i}T_{i}$$

$T_{GM}$: Weighted average melt temperature
$T_{i}:$ Arithmetic mean of the temperature measured at measuring position i
N: Number of measuring positions.

To evaluate thermal homogeneity, the standard deviation *s_therm_* of the melt temperature was calculated by relating the arithmetic mean values of the melt temperatures at the individual measurement positions to the weighted average melt temperature. The variance *s*^2^ and the standard deviation *s_therm_* were determined.

The standard deviations of the different screw configurations were compared to evaluate the thermal homogeneity. The aim was to achieve the lowest possible standard deviation.

## 3. Results

In a previous study [29], the results were examined for the influence of minimal changes in geometry within a screw concept. The evaluation showed that there were no significant differences between the small variations in screw geometry. However, the different screw designs resulted in different melt qualities. For this reason, the discussion and evaluation of the variations in screw geometry will not be part of this work. In the following section, the results of the comprehensive investigation plan are presented as an example.

### 3.1. Evaluation of Material Homogeneity

**Figure 7:**
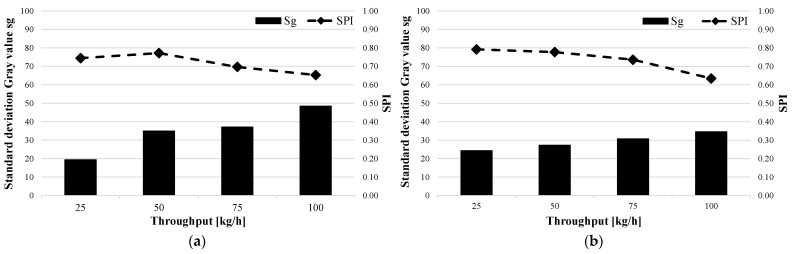
Standard deviations of gray value and SPI as functions of throughput for Lupolen and the two shear and mixing part combinations (**a**) SM1- Lupolen 2420D and (**b**) SM2- Lupolen 2420D.

Figure 7 shows the standard deviation of the gray value and the SPI as a function of throughput for the two shear and mixing part combinations SM1 (a) and SM2 (b) with the Lupolen 2420D. The standard deviation increases with increases in the mass throughput. This is because the melting point is shifted towards the tip of the screw, which prevents the material from being sufficiently homogenized. As can be clearly seen in both figures, the SPI determined from the data of the measuring computer decreases with increases in the throughput. It can also be seen that the shear and mixed-part combination SM2 led to lower standard deviations of the gray value than SM1. From this result, it can be concluded that the spiral shear part (SM1) has a lower homogenizing effect than the Maddock shear part (SM2). However, further investigations should be carried out to validate this thesis.

**Figure 8:**
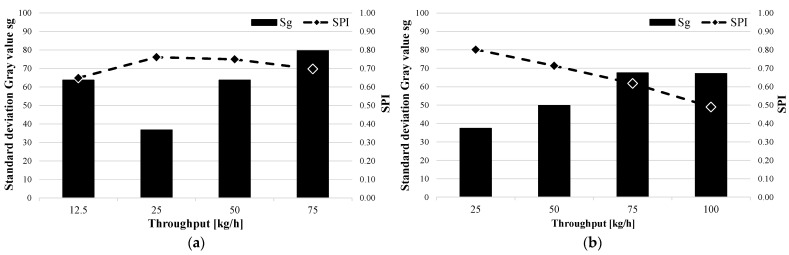
Standard deviations in the gray value and SPI as functions of throughput for the barrier screw BS2 and the three-section screw DZS1: (**a**) BS2–Moplen 420M and (**b**) DZS1–Lupolen 2420D.

In order to support the results from the shear and mixed-part combinations, the standard deviations of the gray value and the SPI for the barrier screws and three-section screws for both materials were also evaluated. Two exemplary test series are shown in Figure 8. On the left, the BS2 barrier screw with the Moplen 420M (a) is shown, and on the right, the DZS1 three-section screw with the Lupolen 2420D is shown. Both the standard deviations of the gray value and the SPI are plotted versus the throughput. It can be clearly seen that the SPI responds to the standard deviation of the gray value. If the standard deviation decreases, the SPI increases; if the standard deviation increases, which reduces the material melt quality, the SPI also decreases. Figure 7a shows that a local minimum of the standard deviation of the gray value occurs at a throughput of 25 kg/h. This is the operating point of the BS2 barrier screw and the Moplen. This local minimum could be due to the fact that at 12.5 kg/h, the back pressure is too low to homogenize the material sufficiently.

### 3.2. Evaluation of the Material Homogeneity

**Figure 9:**
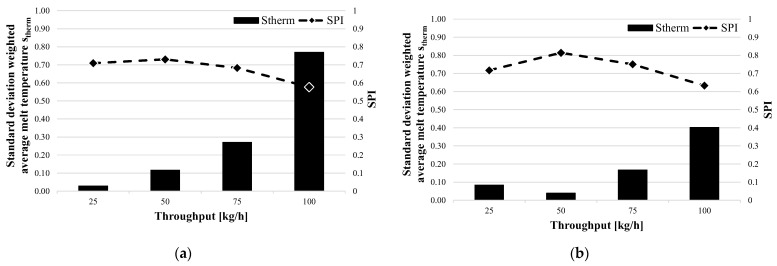
Standard deviations of the weighted average melt temperature and SPI as functions of throughput for SM1 and SM2: (**a**) SM1–Moplen 420M and (**b**) SM2–Moplen 420M.

To investigate the correlation between the SPI and the standard deviation of the weighted average melt temperature, these were also plotted against the throughput. As an example, the standard deviation of the weighted average melt temperature for the two shear and mixing-part, SM1 (a) and SM2 (b), for the Moplen 420 M are evaluated in Figure 9. It can be seen for both elements that the standard deviation increases with increases in the throughput. As previously explained for the material homogeneity, the time required to homogenize the melt both thermally and materially decreases. As previously seen with the standard deviation of the gray value, the standard deviation of the weighted average melt temperature of the SPI also follows the decreasing thermal homogeneity of the polymer melt. 

**Figure 10:**
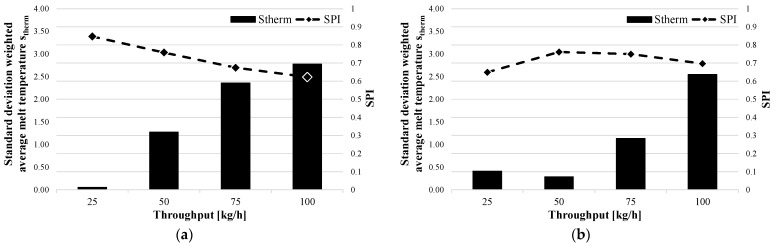
Standard deviations of the weighted average melt temperature and SPI as functions of the throughput for the three-section screw DZS2 and the barrier screw BS2: (**a**) DZS2–Lupolen 2420D and (**b**) BS2–Moplen 420M.

As a verification of this correlation, the results for the other screw designs were also examined. Figure 10 shows the standard deviations of the weighted average melt temperature and SPI versus the throughput. Again, it is clear that the SPI increases as the standard deviation decreases and decreases as the standard deviation increases. Similar to the standard deviation of the gray value, a local minimum can also be observed for the standard deviation of the weighted average melt temperature for the BS2 barrier screw and Moplen (Figure 6b) at a flow rate of 50 kg/h. This observation needs to be validated and explained in further studies.

## 4. Conclusions and Outlook

The objective of this study was to determine if there is a correlation between melt quality and the screw performance index. In this paper, the dependencies of the SPI on the standard deviations of the gray value and the weighted average melt temperature were investigated. The evaluations of the investigations in this paper clearly show that the SPI correlates with the thermal and material homogeneity of the polymer melt. If the standard deviation decreases, the SPI increases because the melt was better homogenized. 

So, it is possible to use the SPI as a target variable for the development of an analytical prediction model. In the prediction model, simulation results obtained using REX 17.1.0 software (a computer-aided extruder design), which was developed at Kunststofftechnik Paderborn (KTP), are to be used as input variables. With the help of artificial intelligence and machine learning, a prediction model will be developed in subsequent work.

## Figures and Tables

**Table 2:** Temperature profiles.

Material/ Screw Concept		Barrel Sections [°C]					Measuring Flange [°C]	Die Sections [°C]
		HZ1.1	HZ1.2	HZ1.3	HZ1.4	HZ1.5	F	WZ
Moplen 420M	Barrier screws BS1 & BS2	70	160	190	220	240	240	240
	Three-section screws DZS1, DZS2, SM1 & SM2	70	170	190	210	220	230	230
Lupolen 2420D	All	70	160	180	200	220	220	220

**Table 3:** Investigation plan.

Throughput [kg/h]			Material	
			Moplen 420 M	Lupolen 2420D
Screw concept	Barrier screws	BS1	12,5, 25, 50, 75	25, 50, 75, 100
		BS2	12,5, 25, 50, 75	25, 50, 75, 100
	Three-section screws	DZS1	12,5, 16, 25, 32,5	25, 50, 75, 100
		DZS2	12,5, 16, 25, 32,5	25, 50, 75, 100
	Shearing and mixing parts	SM1	12,5, 25, 50, 75	25, 50, 75, 100
		SM2	12,5, 25, 50, 75	25, 50, 75, 100

## Data Availability

Not applicable.
